# Structural Fluidity of the Human Immunodeficiency Virus Rev Response Element

**DOI:** 10.3390/v12010086

**Published:** 2020-01-11

**Authors:** Chringma Sherpa, Stuart F. J. Le Grice

**Affiliations:** Basic Research Laboratory, National Cancer Institute, Frederick, MD 21701, USA; chringma@gmail.com

**Keywords:** HIV, Rev response element, chemical footprinting, SHAPE, drug discovery, branched peptides

## Abstract

Nucleocytoplasmic transport of unspliced and partially spliced human immunodeficiency virus (HIV) RNA is mediated in part by the Rev response element (RRE), a ~350 nt cis-acting element located in the envelope coding region of the viral genome. Understanding the interaction of the RRE with the viral Rev protein, cellular co-factors, and its therapeutic potential has been the subject of almost three decades of structural studies, throughout which a recurring discussion theme has been RRE topology, i.e., whether it comprises 4 or 5 stem-loops (SLs) and whether this has biological significance. Moreover, while in vitro mutagenesis allows the construction of 4 SL and 5 SL RRE conformers and testing of their roles in cell culture, it has not been immediately clear if such findings can be translated to a clinical setting. Herein, we review several articles demonstrating remarkable flexibility of the HIV-1 and HIV-2 RREs following initial observations that HIV-1 resistance to trans-dominant Rev therapy was founded in structural rearrangement of its RRE. These observations can be extended not only to cell culture studies demonstrating a growth advantage for the 5 SL RRE conformer but also to evolution in RRE topology in patient isolates. Finally, RRE conformational flexibility provides a target for therapeutic intervention, and we describe high throughput screening approaches to exploit this property.

## 1. Introduction

Conformational “fluidity” of RNA allows it to mediate a variety of biological functions, examples of which include (a) catalyzing cleavage by the hammerhead ribozyme of satellite RNAs and viroids [1]; (b) bacterial riboswitches [2]; (c) RNA thermometers [3]; and (d) tRNA-dependent control of specific aminoacylation and translation regulation [4]. In the case of human immunodeficiency virus (HIV), cis-acting sequences encoded in its (+)RNA genome are central to transcription of the integrated provirus, nucleocytoplasmic transport of unspliced and partially spliced RNAs, initiation of reverse transcription, genome dimerization/packaging, and ribosomal frameshifting [5]. A comprehensive understanding of the structural dynamics of these regulatory elements would be predicted to accelerate development of small molecules [6], oligonucleotides [7], peptide nucleic acids [8] evolved RNA recognition motifs [9], and nucleic acid aptamers [10] as novel therapeutic modalities to complement existing anti-HIV agents. With these objectives in mind, the goal of this review was to highlight conformational flexibility of the HIV-1 RRE (and its HIV-2 counterpart) through a series of experiments that extend in vitro structural analysis to their in vivo outcome in cell culture systems and, finally, in sequential viral isolates in a clinical setting.

**Figure 1:**
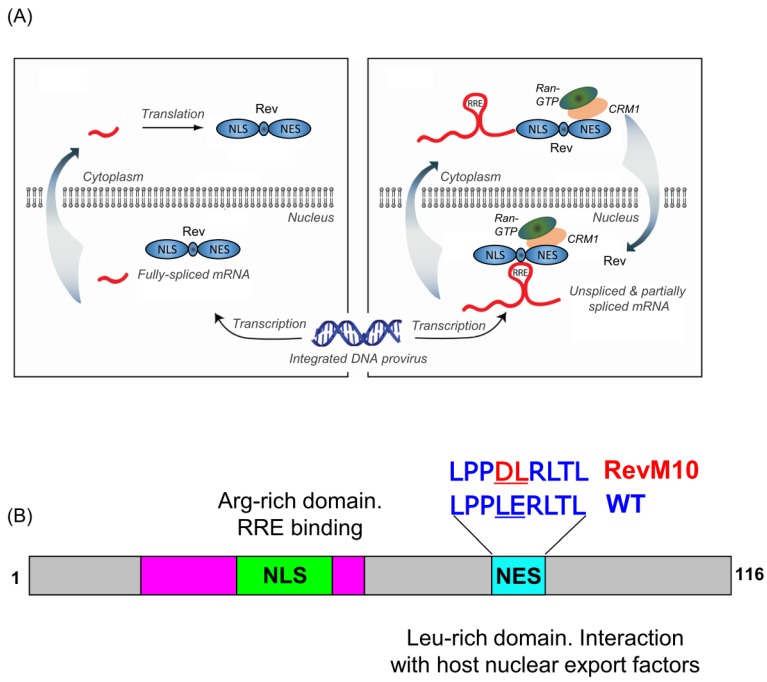
(**A**) Functional requirement of the HIV RRE: Early in the viral lifecycle, fully spliced viral RNAs are exported from the nucleus in a Rev/RRE-independent manner. Among these, Rev mRNA are translated and Rev is imported into the nucleus. In the late phase of the lifecycle, nuclear RRE-containing RNAs recruit Rev and cellular nuclear-export machinery, allowing them to circumvent splicing and transit to the cytoplasm, where they are either translated or packaged into assembling virions (**B**) Organization of the 116 aa HIV-1 Rev and amino acid changes in the trans-dominant M10 variant. NLS; nuclear localization signal, NES, nuclear export signal. Pink areas flanking the NLS represent Rev oligomerization domains.

Figure 1A provides a summary of the functional requirement for the HIV-1 RRE. Productive HIV infection produces three types of viral transcripts, i.e., unspliced, partially spliced, and fully spliced RNAs (http://hivinsite.ucsf.edu). Early in the viral life cycle, fully spliced viral RNAs encoding the regulatory proteins Rev, Tat, and Nef are exported and translated in the cytoplasm. However, the presence of introns in unspliced and partially spliced viral RNAs results in their nuclear retention by host RNA surveillance mechanisms that normally restrict nucleo-cytoplasmic export of intron-retaining mRNAs [11,12]. Later in the life cycle, Rev, through its nuclear localization signal (NLS), is imported into the nucleus [13,14]. The NLS domain is a basic, arginine-rich motif (ARM) that also serves as an RNA-binding domain (RBD) that binds specifically to the RRE (Figure 1B) [15]. Inside the nucleus, Rev binds cooperatively to the RRE present in all intron-retaining viral RNAs through a process involving both protein–protein and protein–RNA interactions [15,16,17,18,19,20,21,22,23,24]. The Rev–RRE complex is recognized by CRM1 and RAN-GTP forming an export competent ribonucleoprotein (RNP) complex [25] allowing unspliced and partially spliced viral RNAs to circumvent host cellular restriction and transit to the cytoplasm, where they are either translated or packaged into assembling virions [26,27,28]. 

A logical first step in understanding molecular details of the Rev/RRE complex was defining the topology of the RRE, an ~350 nt, RNA comprising multiple stem-loops and bulges. Combining computational modeling with chemical and enzymatic footprinting led to the proposal of a 5 stem-loop (SL) structure with a central SL-1 branching into SL-II (the primary Rev binding site), SL-III, SL IV, and SL-V [29,30,31]. The SL-II consists of stem IIA branching out of the central loop and opening into a three-way junction. The junction opens into two stem-loops IIB and IIC [19,24,32,33,34]. In contrast, a 4 SL structure, differing in rearrangement of SL-III and -IV, has been reported [23,35], wherein SL-III and -IV of the 5 SL RRE combine to form a single SL-III/IV off of the central loop. Despite these differences, the 4 SL and 5 SL RRE conformers preserve SL-II topology. Since the majority of these structures were derived from different in vitro probing methodologies, it cannot be ruled out that such differences reflect subtle alterations in buffer probing conditions and are not truly reflective of the biological system. This review summarizes several papers, including analysis of patient isolates that collectively suggest both that HIV-1 and HIV-2 RRE possess sufficient flexibility to adopt alternative conformations, and that for HIV-1, at least, stabilizing these by in vitro mutagenesis confers a growth advantage for the 5 SL conformer. Lastly, we present data suggesting that RRE conformational flexibility might be exploited therapeutically. 

## 2. Resistance to Trans-Dominant RevM10 Therapy Induces a Conformational Change in the HIV-1 RRE

**Figure 2:**
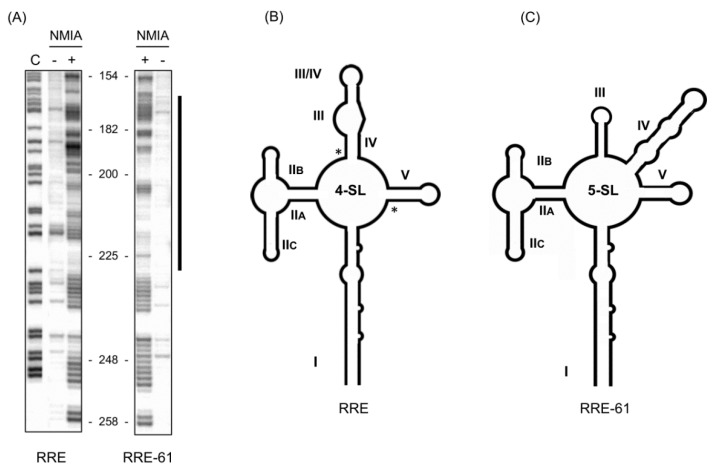
Mutations conferring resistance to trans-dominant RevM10 therapy induce a conformational change in the HIV-1 RRE. (**A**) Structural probing of the wild-type RRE (left) and RevM10-resistant RRE-61 (right). (**C**) Control DNA sequencing lane from which nucleotide numbering was derived. Designations − and + refer to untreated and NMIA-treated RNA, respectively. Major alterations in chemical reactivity are indicated by the bar. (**B**,**C**) Cartoons depicting SHAPE-derived conformations of wild-type RRE and RRE-61. SL, stem-loop. Positions of RRE point mutations, inducing RevM10 resistance, are indicated by asterisks in (**B**). Modified from Reference [47].

The prototype Rev (NL4-3) from HIV clade B is a ~18 kD 116 amino acid phospho-protein [36]. Thee Rev protein comprises several well-characterized functional domains central to Rev-RRE-mediated nucleocytoplasmic transport of unspliced and partially spliced mRNAs [13]. One of these, aa 34–50 constitute the arginine-rich nuclear localization signal (NLS), the primary contact point to the RRE. The NLS is flanked by oligomerization domains that are required for Rev multimerization. Oligomerization is mediated mainly by Leu 12, Val16, Leu18, Leu55, and Leu60 [37]. Towards the carboxy-terminal is a leucine-rich nuclear export signal (NES) (aa 75–84) that mediates interactions with host nuclear export factors (Figure 1B, [38]). During a functional delineation study of Rev by site-directed mutagenesis, Malim et al. [39] constructed a defective variant of Rev, designated RevM10, by replacing two critical NES residues (Figure 1B). When expressed in transfected cells, this mutant Rev protein successfully inhibited the function of its wild-type counterpart. That RevM10 serves as an effective trans-dominant inhibitor of Rev function was further validated by others [40,41,42,43,44] thereby catapulting RevM10-based gene therapy into Phase I and II clinical trials [45]. However, a subsequent study by Hamm et al. [46], involving passage of HIV-1 in a T-cell line constitutively expressing RevM10, reported rapid emergence of a RevM10-resistant virus [46]. Surprisingly, escape mutations were not associated with Rev, but rather the RRE. The same study demonstrated that only two silent mutations (with respect to viral envelop protein) outside the primary Rev-binding region of the RRE, namely, G164 > A164 (at the base of SL-III/IV) and G245 > A245 (in the central loop) sufficed to confer RevM10 resistance, suggesting a conformational change in the RRE was responsible. With this in mind, RRE structures of wild-type and RevM10-resistant HIV variants containing these two RRE mutations (RRE-61) were examined by selective 2′-OH acylation monitored by primer extension (SHAPE) [47]. In this study, the chemical reactivity profile of the wild-type RRE (Figure 2A) predicted the 4 SL conformer (Figure 2B). In contrast, the two silent RRE-61 mutations, inducing RevM10 resistance, introduced significant alterations in chemical reactivity over ~60 nt (Figure 2A), deconvolution of which predicted the 5 SL conformer (Figure 2C). Constructing individually-mutated RRE variants, G164 > A164 (at the base of SL-III/IV) and G245 > A245 (in the central loop), allowed their contribution to be evaluated, where a combination of in vitro replication kinetics, non-denaturing polyacrylamide gel electrophoresis, and chemical footprinting indicated the G245 > A245 mutation sufficed to mimic the structure and activity of RRE-61. Interestingly, in this study, RevM10 resistance could not be attributed to differential/reduced Rev binding to RRE-61. Therefore, it is likely that the stable SL-IV structure in the 5 SL RRE61 provides binding sites for cellular or viral factors that mediate RevM10 resistance (see later). Nevertheless, the study by Legiewicz et al. [47] demonstrated a considerable degree of conformation flexibility inasmuch as a relatively modest nucleotide change in the RRE central loop had a major impact on its topology.

## 3. Structural Conformers of the Wild-Type HIV-1 RRE

Since the discovery of Rev [48,49] and the RRE [26,27,28,50] about 30 years ago, a wealth of studies on the HIV-1 Rev-RRE system have advanced our understanding of the structural details of the system. A consensus secondary structure of the RRE, however, has been lacking. Although the minimal size of a functional RRE was initially reported to be 234 nt [28], later studies [23,31,32] indicated that a fully functional RRE is ~350 nt. Early studies on RRE secondary structure were performed with the subtype B HXB2 234 nt RRE [29,30] which was reported to assume a 5 SL structure. As NL4-3 increasingly become the standard HIV molecular clone studied in laboratories, subsequent structural studies of the RRE were performed mostly on this isolate. Interestingly, studies on the 351 nt NL4-3 RRE also supported a 4 SL conformer [23,35]. It was then speculated that the presence of a longer stem-loop I used might have facilitated the alternative structure. However, contrary to this speculation, Watt et al. [31] subsequently showed that the full-length RRE of RNA purified from HIV-1 NL4-3 virions adopts a 5 SL conformation. This study, therefore, showed that at least, in NL4-3 RRE, the longer stem-loop I was not the determinant of the alternative structure. This finding was further supported by another study that used SHAPE to show that an in vitro transcribed 232 nt NL4-3 RRE formed the alternative 4 SL structure [47]. Besides HXB2 and NL4-3, the secondary structure of another HIV-I molecular clone (ARV-2/SF2) RRE (354 nt), differing from NL4-3 sequence at 13 nucleotides, has also been reported to adopt a 5 SL like conformation. This variant differs from the canonical 5 SL structure in that the nucleotides to the left of the top stem region of SL I/I’ base pair with nucleotides from the central loop, bridging SL-IV and SL-V and forming a stem region which opens into individual SL-IV and SL-V. 

**Figure 3:**
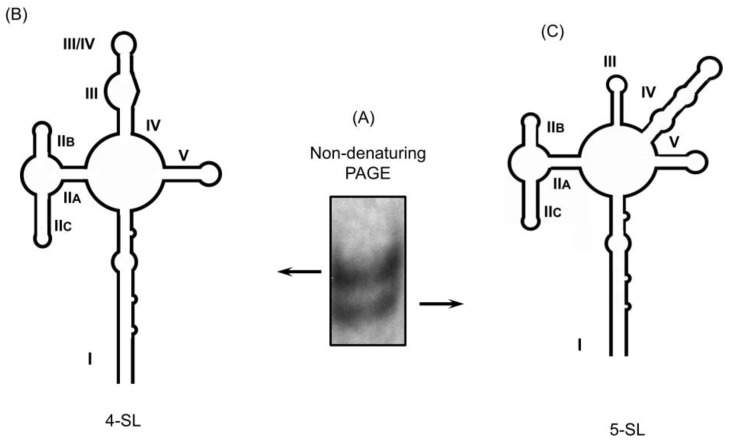
The HIV-1 RRE exists in a conformational equilibrium. (**A**) Following extended non-denaturing PAGE, slow and fast migrating RRE conformers were observed. Subjecting these RNAs to in-gel SHAPE defines these as 4 SL (**B**) and 5 SL conformers (**C**). Note that, despite their conformational heterogeneity, the topology of SL-II, the primary Rev binding suite, is preserved. Modified from Sherpa et al. [52]. The 232 nt HIV-1 RRE RNAs appended with a 3′ structure cassette were prepared for analysis by in vitro transcription.

Since chemical probing techniques used in these studies provide ensemble-average structural information, it could not be excluded that RRE structural heterogeneity might give rise to such discrepancies. In-gel probing, in contrast, offers an alternative strategy to examine conformationally heterogeneous RNAs, providing that they can be separated by non-denaturing strategies. As an example, Kenyon et al. [51] applied in-gel SHAPE to define the structure of monomeric and dimeric species of the HIV-1 packaging signal RNA, supporting a structural switch model of RNA genomic dimerization and packaging [51]. In light of (i), HIV-2 RRE conformational heterogeneity (see later) and (ii), the observation that a single nucleotide alteration sufficed to stabilize the 5 SL HIV-1 RRE conformer [47], we showed that by extended non-denaturing polyacrylamide gel electrophoresis that the HIV-1 RRE could be resolved into two closely migrating species (Figure 3A) [52]. Subsequent in-gel SHAPE verified the slower migrating RNA as the 4 SL conformer and the faster migrating RNA as the 5 SL conformer (Figure 3B,C, respectively), suggesting that in vivo, the wild-type HIV-1 RRE could exist in a conformational equilibrium. 

**Figure 4:**
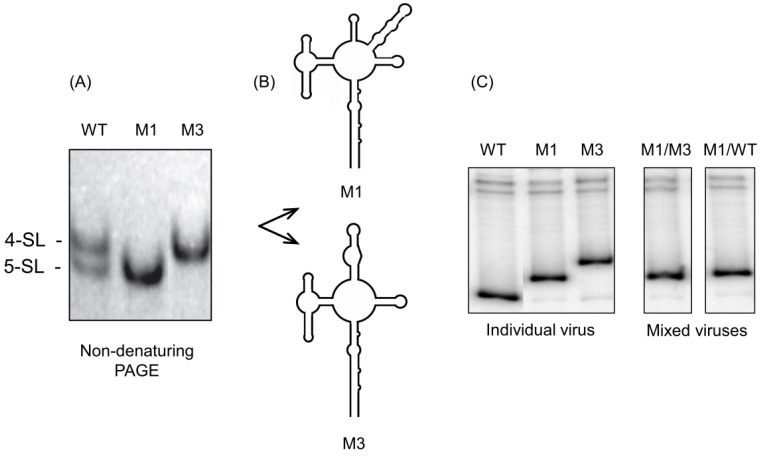
Alternative HIV-1 RRE conformers promote different rates of virus replication. (**A**) Conformer construction (see text). (**B**) Chemical acylation (SHAPE) confirms a 5 SL conformation of mutant M1 and a 4 SL conformation of mutant M3. (**C**) Heteroduplex tracking analysis. In both M1/wt and M1/M3 mutant co-infections, the stabilized 5 SL M1 conformer displays a replicative growth advantage. The full experimental background is provided in Reference [52].

To investigate the function of these alternate HIV-1 RRE conformers, Sherpa et al. [52] created stable 4 SL and 5 SL RRE variants by in vitro mutagenesis to determine their Rev-RRE activity. The RRE Mutant M1 was predicted to disrupt base pairing at the base of the combined SL-III/IV structure in the 4 SL conformer but maintain base pairing in SL-IV in the 5 SL variant and, thus, likely to adopt only the latter structure. Conversely, the mutant RRE M3 was expected to disrupt the base pairing in both SL-III and SL-IV of the 5 SL structure but keep the combined SL-III/IV intact and, thus, likely adopt only a 4 SL conformation. As illustrated in Figure 4A,B, these predictions were borne out experimentally since the initial RRE population was resolved into two stable conformers. The SHAPE analysis indicated that the mutant RREs preserved the structure predicted by mutagenesis. More importantly, electrophoretic mobility shift experiments indicated that Rev binding to the mutant RREs was largely unaffected, demonstrating that their global topology had not been affected by mutagenesis. Growth competition assays were next performed in a T-cell line (SupT1) to assess whether these RRE conformers conferred a selective growth advantage. In this heteroduplex tracking analysis strategy [53], viruses with a different RRE were added to the same culture of SupT1 cells at an equal multiplicity of infection (MOI) and allowed to replicate and spread throughout the cultures for several days, after which cellular DNA was recovered. Relative amounts of integrated pro-viral DNA produced by each virus was measured using a PCR-based heteroduplex tracking. Since sequence mismatches within the wt/M1 and wt/M3 heteroduplexes caused them to migrate differently from each other and the perfectly wt/wt homoduplex, each heteroduplex could be resolved by native gel electrophoresis. Figure 4C indicates that the stable 5 SL RRE conformer displayed a selective growth advantage over its stable 4 SL counterpart as well virus encoding the wt RRE. Supporting this observation, HIV gag/pol expression assays demonstrated that the stabilized 5 SL conformer was functionally superior to wt and the 4 SL RRE. Thus, structural plasticity of the RRE promoting similar [54] or different levels of Rev-RRE function demonstrates how the Rev-RRE regulatory axis might function as a “replication rheostat” rather than a simple on/off switch.

## 4. Conformational Flexibility of HIV-1 RRE SL-I and Rev Sequestration

Although detailed structural data are available for the HIV-1 RRE in either the absence or presence of Rev [18,20,55], understanding how the Rev-mediated nuclear export complex assembles has proven a challenge. In the model proposed by Frankel and co-workers, initial Rev binding to SL-IIB promotes its recruitment to an additional secondary site on SL 1, with at least six copies of Rev ultimately driving formation of the functional ribonucleoprotein complex [19].

**Figure 5:**
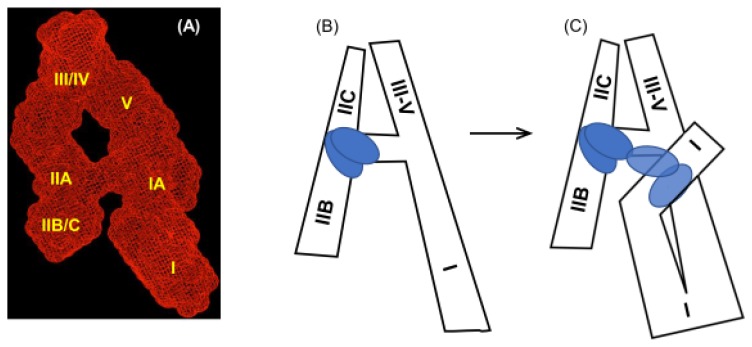
Conformational flexibility of HIV-1 RRE SL-I contributes towards assembly of the Rev-mediated export complex. (**A**) Molecular envelope of the RRE RNA, drawn in mesh and derived by SAXS [32]. The spatial resolution of the envelope is 21 A°. (**B**) Cartoon representation of the RRE, depicting assembly initiating via a single nucleation point in SL-II for two Rev molecules (blue). (**C**) Through an SL-I conformational change, “coupling” of SL-I and SL-II Rev-binding sites promote a tetrameric intermediate complex proposed to serve as a specificity checkpoint. Rev and the RRE could thereafter simultaneously sample a number of interaction conformations until an optimal binding state for Crm1 binding and nuclear export is attained.

In this model, an additional role for SL-I beyond providing the accessory Rev-binding site had not been considered. Using time-resolved SHAPE and SAXS, Bai et al. [32] demonstrated that SL-I flexibility makes an important contribution to the formation of the export complex, illustrating that Rev binding affects chemical acylation levels/structure at three distinct regions (I, II, II) in the RRE. Region I maps to the high affinity Rev binding site and the stem II three-way junction. Region II covers the central loop and the top region of SL-I that includes the previously identified secondary Rev binding site [19], while Region III lies at the center of SL-I of the 351 nt RRE. Figure 5A depicts the SAXS-derived A-like structure of the RRE reported by Feng et al. [55] with an RNA construct whose SL-I was truncated. In this model, the maximum diameter of the RRE was calculated ~195 A°. Surprisingly, this value did not change when an RRE containing the full-length SL-1 was re-examined by SAXS [32], suggesting that rather than adopting an extended “tail”, SL-I folds into and interacts with the RRE core to adopt a compact structure. Proof of this notion was provided by hybridizing an antisense oligonucleotide to either of the interacting partners. In both cases, an increase in the maximum diameter ~300 A° was observed, suggesting disruption of the long-range SL-1-mediated interaction. Based on their findings, Bai et al. [32] propose a model where a Rev dimer binds to Region I of a pre-organized RRE which is immediately followed by binding of another Rev dimer in Region II. This induces tertiary long-range interaction between Region III and the central loop region, exposing a cryptic Rev binding site in SL-I, to which additional Rev molecules can bind [23] (Figure 5B,C). While once more demonstrating remarkable flexibility of the RRE, data from Bai et al. [32] raises the intriguing notion of developing multi-dentate ligands to target long-range interaction critical to Rev/RRE assembly as a therapeutic strategy. Data from a later section address this possibility.

## 5. Interchanging HIV-1 RRE Conformers in Patient Isolates

**Figure 6:**
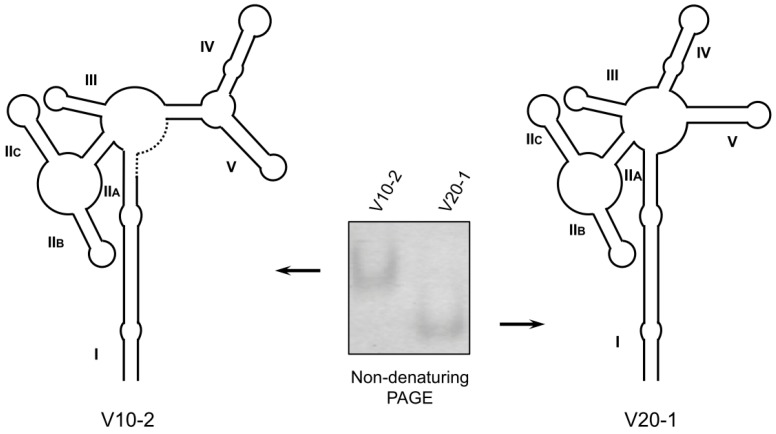
Patient-derived HIV-1 RREs from early and late time-points post-infection exhibit different secondary structures. Secondary structures of V10-2 RRE (an early isolate, (**left**) and V20-1 RRE (a late isolate, (**right**) determined by SHAPE-MaP. (**Center)** differential migration rate of V10-2 and V20-1 RRE, following non-denaturing PAGE and UV shadowing, is suggestive of alternate conformers/ Adapted from Sherpa et al. [59].

Several lines of in vitro evidence discussed thus far suggest the RRE as a dynamic structure capable of existing as a mixture of conformers or assuming alternate conformations in response to minor nucleotide changes (e.g., RRE-61). Such observations raise the question whether this might also occur in a clinical setting in the course of HIV infection. Indeed, nucleotide changes over the course of virus infection have been linked with enhanced RRE activity and a more rapid CD4 decline [56], while decreased Rev activity has been linked to slower disease progression and reduced susceptibility to T-cell killing [57]. Efforts to develop new therapeutic interventions directed at the Rev/RRE axis would likely benefit from studies of its structural and functional evolution in the course of natural infection. To address this, Sloan et al. [58] examined evolution the Rev/RRE axis in the blood plasma of a single patient using samples collected from initial detection of p24 antibodies within 6 months of infection (designated visit 10 or V10-2) through the subsequent 6 years of infection in the absence of antiretroviral therapy (designated V20-1). Functionally, this study demonstrated that V20-1 RRE promoted Rev multimerization at a lower Rev concentration than its V10-2 counterpart. In a follow-up study, a structural analysis of the V10-2 and V20-1 RREs was undertaken by Sherpa et al. [59]. As shown in Figure 6, non-denaturing gel electrophoresis differentiated among these two RREs based on their migration properties, suggesting alternate conformers. Chemical probing analysis indicated that a portion of the V10-2 central loop between SL-III and SL-IV paired with nucleotides from the upper stem of SL-I, forming a stem that bridges the central loop and SL-IV and -V. In contrast, V20-1 RRE formed the canonical 5 SL structure, with SL-I to SL-V radiating directly from the single-stranded central loop. This is the first detailed long-term study to examine longitudinal RRE evolution in a patient following infection, highlighting that selection pressures impart an influence on the RRE sequence, with a tendency toward increased functional activity. Increased activity has been be explained by large-scale conformational changes within the RRE and a decrease in base-pairing stability at the initial Rev binding site of SL-II. Although selective pressure on the HIV *Env* gene during disease progression in terms of immune evasion and replication efficiency may well be contributing factors, functional differences in Rev-RRE activity likely also contribute to viral fitness.

This study, also for the first time, showed experimentally that structural fluidity exists in the SL-II region of primary HIV isolates which can modulate Rev-RRE activity. A more recent paper [60] further explored the structural flexibility of RRE SL-II region using NMR to highlight that in vitro synthesized wt (NL4-3) SL-II exists in dynamic equilibrium of three different conformers which includes two non-native excited states (ES1 and ES2) that remodel key structural elements required for Rev binding and one ground state (GS). These ES populations constitute around 20% of the SL-II structural ensemble and bound Rev peptides with 15 to 80 fold weaker affinity. Such studies highlight the need to consider structural flexibility of SL-II regions in developing anti-HIV therapeutics targeting the RRE as traditional approaches that rely on high throughput screening and/or rational design of small molecules/peptides/agents that bind to the GS RRE II. Agents that lock the RRE in the less active ES forms should therefore be explored as new avenues for anti-HIV drug design.

It is also important that structural flexibility of regions of the RRE outside the primary Rev binding site be considered during anti-HIV drug design. A good example of this notion is reflected in development of drug resistance against ENF (enfutivirtide or T20), the first fusion inhibitor used for HIV treatment. T20 acts by binding to a region of gp41 subunit of HIV Env and has been reported to select for secondary mutations in Rev and the RRE [61]. The primary mutations associated with ENF resistance were located within the ENF target region and map to gp41 aa 36–45 which lies within the RRE. Secondary mutations were found to restore the RRE structure predicted to be disrupted by the primary mutations. Such “structure conservation mutations” were observed in SL-IIC [61] and SL-III [62], underscoring the importance of conformational fluidity beyond the primary Rev binding site. A thorough molecular understanding of the various alternative RRE conformers in primary isolates will therefore be pivotal in designing more effective anti-HIV drugs that delay/prevent the onset of RRE structural flexibility-mediated drug resistance.

## 6. Conformational Changes Underlying “Maturation” of the HIV-2 RRE

**Figure 7:**
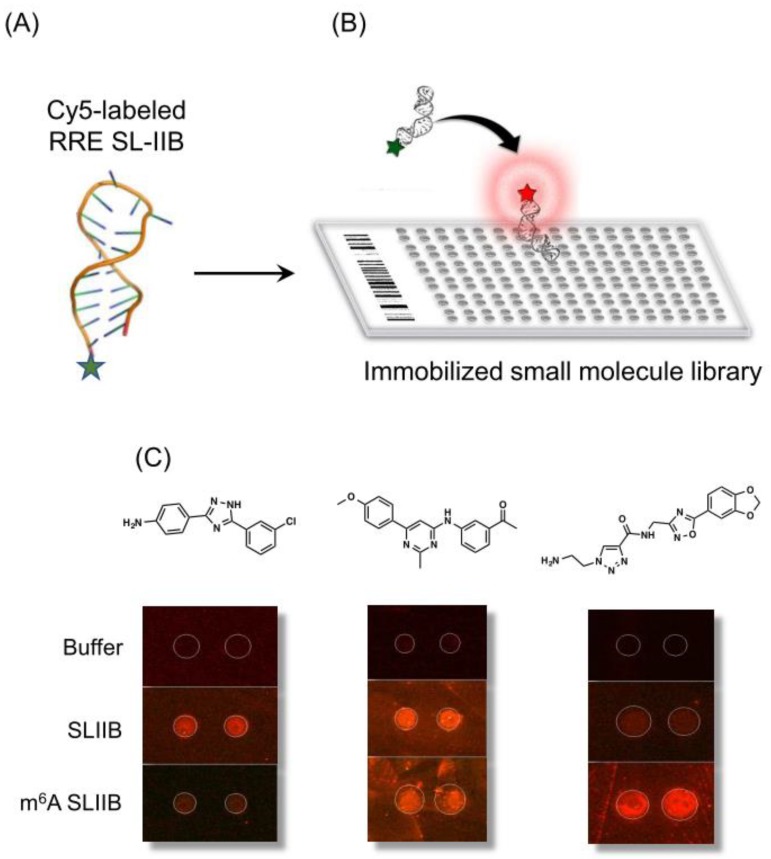
Time-dependent conformational rearrangement of the HIV-2 RRE. Analyses were performed on a 216 nt RRE derived from HIV-2_ROD_ by in vitro transcription. (**A**) Native gel electrophoresis as a function of incubation time indicates the HIV-2 RRE comprises a mixture of “open”, “intermediate”, and “closed” conformers (**A**–**C**, respectively) at short incubation times and whose ratio varies with time, with the closed conformer ultimately predominating. (**B**) SHAPE-derived conformations of the open, intermediate, and closed HIV-2 RRE forms, respectively. Secondary structural motifs are indicated and color-coded as follows: SL-I, red; S-IIA, dark green, SL-IIB, -IIC and adjacent connecting loops, magenta; SL-III, yellow; SL-IV, blue; SL-V, orange. Modified from Reference [64].

An intriguing issue is whether observations and models suggesting structural fluidity are unique to the HIV-1 RRE or whether its’ HIV-2 counterpart is likewise conformationally heterogeneous. Early mutational studies of the HIV-2 RRE [63] indicated that (i) the interaction with its cognate Rev was more dependent on maintenance of secondary structure than primary nucleotide sequence and (ii) HIV-2 RRE structures permitting interaction with HIV-1 Rev, while coinciding with those required for HIV-2 Rev binding, were dissimilar in structure and nucleotide sequence. Prior to performing HIV-2 RRE characterization by SHAPE, data from Figure 7A raised a formidable challenge, since in the absence of any binding partner this too displayed unexpected conformational flexibility. Although denaturing polyacrylamide gel electrophoresis indicated a single RNA species following in vitro transcription, subsequent non-denaturing electrophoresis identified three conformers that gradually “coalesced” into a single species upon prolonged renaturation [64]. Since SHAPE requires that the target RNA adopt a uniform structure in solution [65], understanding this unexpected stepwise HIV-2 RRE folding required a mathematical model to be developed that extracted the contributions of individual conformers from ensemble chemical reactivity values. The model makes two assumptions. Firstly, it assumes that each ensemble SHAPE reactivity value obtained from a population of RNA conformers equals the sum of reactivity values of the contributing conformers, weighted according to their fractional contribution to the total RNA population. Secondly, if ensemble reactivity values and fractional contributions of individual conformations changed with differing conditions (e.g., folding time), and these values could be determined for a number of conditions equal to or greater than the total conformer content of the mixture, specific chemical reactivity values for each conformer could be mathematically derived. By adopting this strategy, the HIV-2 RRE folding program depicted in Figure 7B was proposed.

The most significant differences among these structures involved the central junction, changes within which affect positioning of the substructures relative to each other. Transition from the open to intermediate conformation requires several concerted substructure translations/rotations, most notably rotation of SL-IIB/IIC, caused by base pair formation in S-L IIA. Pairing of the bridge helix defines the subsequent transition from the intermediate to closed conformer, which also involves inward rotation of the SL-IV/V substructure, folding of the SL-IIB apical loop toward the central junction and formation of mutually stabilizing contacts between SL-IIB and SL-V. The SL-IIB is arranged orthogonally to SL-IIC and coaxially with SL-I, consistent with assembly models, whereby Rev initially binds to high affinity sites on SL-IIB and SL-I, then multimerizes linearly along an SL-IIB/SL-I axis. Given the close contact between SL-IIB and SL-V in the closed conformer, rotational/translational flexibility of the SL-IV/V substructure would be required to create space for Rev binding to SL-IIB.

## 7. Targeting RRE Conformations: the HIV Epitranscriptome.

Over several decades, post-transcriptional chemical modifications that impact RNA metabolism, function, and localization have been recorded, the most common associated with tRNA and ribosomal RNA. Of the >100 post-transcriptional modifications identified to date, N^6^-methyladenosine (m^6^A) has been the most extensively studied with respect to its importance in health and disease [66]. Although the functional role remains to be fully elucidated, a role for post-transcriptional modification of the retroviral RNA genome was first proposed from studies by Kane et al. [67]. Three recent studies have established m^6^A modification of the HIV-1 genome [68,69,70], of which Lichinchi et al. [69] have suggested that methylation of conserved adenosines in RRE SL-II (A7877 and A7883) enhanced Rev binding and influenced nuclear export of viral RNA.

**Figure 8:**
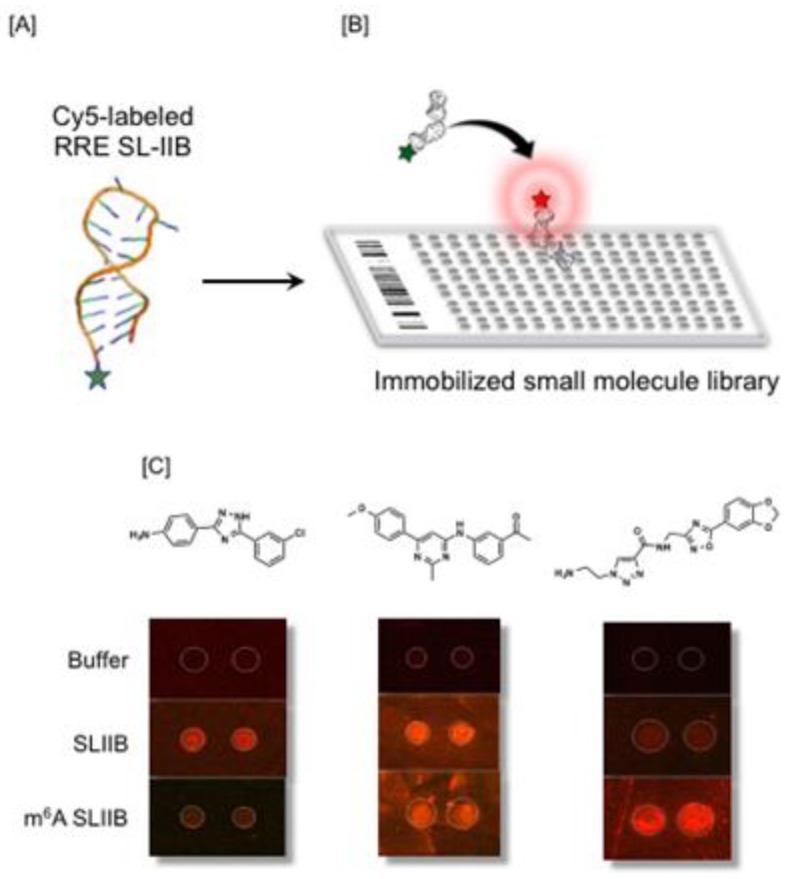
Screening for small molecules that recognize m^6^A-modifed HIV-1 RRE SL-IIB. (**A**) A synthetic RNA fragment harboring the m^6^A modifications reported in SL-IIB is labeled with Cy5 on its 3′ terminus. (**B**) Labeled SL-II RNA was flowed over microtiter plates containing covalently immobilized small molecules. Binding of RRE SL-II RNA to candidate ligands was recorded via a fluorescence signal. (**C**) HTS screening suggests specificity of small molecules for unmethylated (left) and methylated SL-IIB (right). The central panel highlights a ligand that recognizes both SL-II forms.

Of possible roles ascribed to post-transcriptional modification, alterations to RNA structure to up- or downregulate interactions with either host or viral proteins would seem likely [71]. An extension of this notion would be the potential to develop small molecules that specifically recognized m^6^A-induced structural alterations within the RNA genome. As a step in this direction, a small molecule microarray (SMM) strategy was developed to allow facile and rapid screening for RNA-binding chemotypes [72,73,74]. The SMM strategy is outlined in Figure 8A, wherein a short, structured RNA (40–60 nt), appended with a fluorophore, is flowed over a library of covalently immobilized small molecules. Ligands interacting with the RNA are recorded as a fluorescent signal (Figure 8B). To determine whether differential recognition of modified RNA could be achieved, unmethylated and m^6^A-modified RREs were screened. As indicated in Figure 8C, chemotypes specific for SL-IIB and m^6^A SL-IIB were identified, in addition to a third class that recognized both RNAs. Although recent study of Chu et al. [75] employing several biochemical and biophysical strategies concluded that the stability, structure, and dynamics of RRE SL-IIB are only marginally affected by m^6^A modification, SMM screening implies that these might be sufficient for selective ligand recognition. While high throughput screening strategies for RNA are in their infancy, data reported here suggests the notion of targeting viral epitranscriptomes should not be overlooked.

## 8. Exploiting RRE Conformational Flexibility with Branched Peptides

The unique 3D architecture of RNA, often achieved by assuming a combination of local structures including bulges, stem-loops, pseudoknots, and turns, presents an attractive therapeutic opportunity. While exemplified by antimicrobial agents, such as aminoglycosides, macrolide, oxazolidinone, and tetracycline, that interfere with ribosomal RNA function, the field of small molecule RNA therapeutics, despite advances in high throughput screening technologies, has not fully matured and would benefit from innovative strategies.

**Figure 9:**
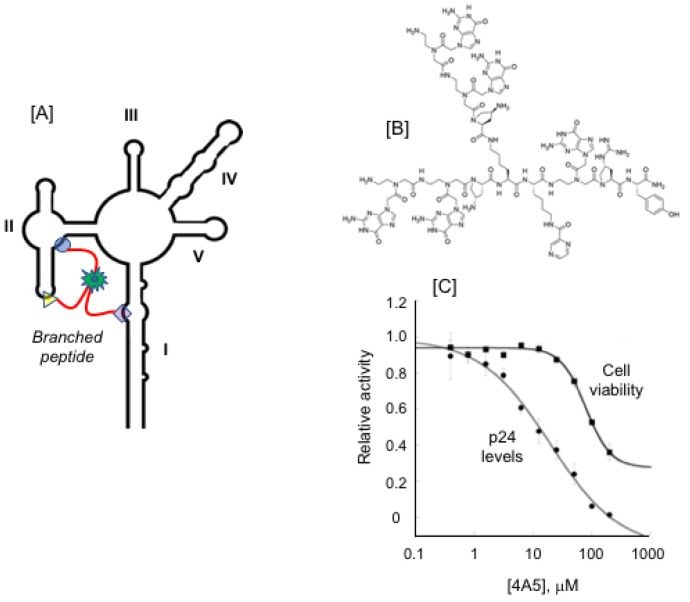
Restricting RRE conformational flexibility with multivalent branched peptides. (**A**) Cartoon depicting the branched peptide strategy, i.e., binding in a multivalent fashion to enhance affinity and selectivity toward the RNA target. (**B**) Structure of branched peptide 4A5. (**C**) Inhibition of Rev-RRE function in vivo using HEK 293T cells transiently transfected with a Rev-expressing plasmid and a CMV promoter-driven GagPol-RRE plasmid. Modified from Dai et al. [77].

As an alternative strategy that considers molecules of intermediate size, branched peptides offer the possibility of supporting multivalent interactions with different regions of a highly flexible RNA such as the RRE (Figure 9A) and would be predicted to enhance their selectivity and affinity. Support for branched peptides was provided by Bryson et al. [76], whose on-bead screening of a 4000 molecule library identified ligands that spanned the bulge and apical loop of HIV-1 TAR RNA with binding affinities in the low micromolar range. Subsequently, to enhance selectivity/affinity Dai et al. [77] synthesized a ~46,000 compound on-bead library of branched peptides composed of unnatural amino acids (Figure 9B). These included L-guanidinoproline and D-aminoproline as electrostatic mimics of arginine and lysine, respectively, 1-naphthalene to promote π— π stacking with nucleobases and pyrazine which acts as a hydrogen bond donor/acceptor. High throughput screening identified the branched peptide 4A5 (Figure 8B) as a high affinity ligand for a synthetic RRE SL-IIB mimic (K_d_ = 0.88 ± 0.02 μM). The SHAPE analysis indicated that at a 1:1 RNA/4A5 ratio, nucleotides of a loop constituting the secondary SL-1 Rev binding site were protected from modification, while at a higher branched peptide/RNA ratio, nucleotides within SL-II became refractory to acylation. From this study, roles for 4A5 in occupying the active Rev binding sites and/or acting allosterically to prevent the SL-I-driven conformational change in the RRE were proposed. Although the exact mechanism remains to be elucidated, the SHAPE data lend credence to the notion of multivalent binding of branched peptides to the RRE. Finally, in the same study, 4A5 was demonstrated to inhibit Rev-RRE function in cell culture using HEK 293T cells transiently transfected with a Rev-expressing plasmid and a CMV promoter-driven GagPol-RRE plasmid (Figure 9C).

## 9. Cellular Factors Interacting with the Rev and the RRE

While this review has concentrated on the dynamic nature of the HIV-1 and HIV-2 RREs, it is important to emphasize that concerted binding of various cellular factors is required for Rev-RRE function, most of which promote shuttling of Rev and RRE across the nuclear membrane. Two key factors are Crm1 [25,78,79,80] and importin β [81,82]. Crm1, or Exportin-1, is a member of the β-importin family of transport receptors and has been demonstrated to specifically interact with many proteins containing NES domains. Normally employed for the nucleocytoplasmic transport of proteins, snRNAs and rRNAs, the Crm1 pathway is hijacked by HIV to facilitate export of intron-retaining RNAs. Crm1 serves as an adaptor for binding of Ran-GTP [83,84,85] and nucleoporins such as Rip/Rab [86] to Rev, forming a functional export complex that ferries the Rev/RRE RNP across the nucleopore complex into the cytoplasm. In the cytoplasm, RanGAP1 and RanBP1 hydrolyze Ran-GTP to Ran-GDP, releasing Crm1 from the Rev/RRE complex [86]. Importin-β then binds to the Rev NLS region, facilitating displacement of the RRE for translation of viral transcripts. The resulting Rev/importin-β complex interacts with Ran-GDP, allowing translocation of the Rev complex into the nucleus. Inside the nucleus, RCC1 catalyzes conversion of Ran-GDP to Ran-GTP, leading to dissociation of Rev from importin-β [87]. This dissociation unmasks the Rev NLS domain, promoting interaction of its NLS/RBD region with the RRE. Several members of the DEAD box RNA helicase family, such as RHA [88], DDX3 [89], DDX1 [90,91], DDX5 [92] and DDX24 [93], have also been reported as cellular co-factors of Rev, although their precise role in the Rev/RRE pathway remains to be elucidated. Most of these are nucleocytoplasmic shuttling proteins some of which have been implicated in promoting HIV genome packaging, restriction of Rev function in astrocytes, premature release of intron-retaining HIV transcripts from the splicing machinery. Similarly, cellular factors such as eIF-5A, SF2/ASF, B23, p32, Sam68, and ATM kinase have been proposed to modulate Rev/RRE function, though their roles are either unclear or controversial [38,94].

## 10. Conclusions and Outlook

Although the role of the RRE in directing nucleocytoplasmic transport of unspliced and partially spliced viral RNAs has been unchallenged for over three decades, the structural flexibility that mediates these steps in HIV replication is only now coming to light, in many cases, following the development of new RNA probing strategies. For example, the generally accepted notion that RRE SL-I played a structurally “passive” role has now been challenged by chemical and biophysical techniques suggesting it folds into the central RRE core, possibly as a check for Rev loading. By extending early data on the structure of RRE-61, we now have evidence that the wild-type RRE may, in fact, exist as an equilibrium of conformers, each of which confers distinct growth properties in cell culture that may extend as far as their evolution in HIV-infected individuals. One might speculate that RRE structural fluidity would confer growth/fitness advantage to the virus by acting as “replication rheostat”, regulating the level of HIV gene expression by allowing RRE to adopt various functional states. A less active RRE would shield HIV from a functional host immune system during early stages of infection, allowing the virus to establish itself within the host. Conversely, highly active RREs would promote a higher rate of virus replication without CTL killing in immunocompromised individuals. Unlike mechanisms that affect overall transcription and translation, down-modulation of gene expression by less active RRE conformers primarily affects viral structural protein expression and is not expected to affect HIV Nef expression. Thus, this mechanism would allow the immune evasion activities of Nef to persist, while the lower structural protein levels would make the infected cells less susceptible to CTL killing, As we uncover additional aspects of RRE conformational dynamics, exploiting this vis-à-vis developing novel high throughput screening strategies, exemplified here with branched peptides and small molecules that recognize the viral epitranscriptome, promises to open new and exciting avenues for targeting viral RNA genomes.
